# Extracellular vesicle treatment partially reverts epigenetic alterations in chronically ischemic porcine myocardium

**DOI:** 10.20517/2574-1209.2023.103

**Published:** 2023-11-03

**Authors:** Mark Broadwin, Ghazal Aghagoli, Sharif A. Sabe, Dwight D. Harris, Joselynn Wallace, Jordan Lawson, Ashok Ragayendran, Alexey V. Fedulov, Frank W. Sellke

**Affiliations:** 1 Division of Cardiothoracic Surgery, Department of Surgery, Cardiovascular Research Center, Rhode Island Hospital, Alpert Medical School of Brown University, Rhode Island Hospital, Providence, RI 02909, USA.; 2 Center for Computation and Visualization, Brown University, Providence, RI 02912, USA.; 3 Center for Computational Biology of Human Disease, Brown University, Providence, RI 02912, USA.; 4 Division of Surgical Research, Department of Surgery, Rhode Island Hospital, Alpert Medical School of Brown University, Rhode Island Hospital, Providence, RI 02909, USA.

**Keywords:** Methylation, methylome, myocardial ischemia, cardiovascular disease, epigenetics

## Abstract

**Introduction:**

Research has shown epigenetic change via alternation of the methylation profile of human skeletal muscle DNA after Cardio-Pulmonary Bypass (CPB). In this study, we investigated the change in epigenome-wide DNA methylation profiles of porcine myocardium after ischemic insult in the setting of treatment with extracellular vesicle (EV) therapy in normal *vs*. high-fat diet (HFD) pigs.

**Methods:**

Four groups of three pigs underwent ameroid constrictor placement to the left circumflex artery (LCx) and were assigned to the following groups: (1) normal diet saline injection; (2) normal diet EV injection; (3) HFD saline injection; and (4) HFD EV injection. DNA methylation was profiled via reduced-representation bisulfite sequencing (RRBS) and compared using a custom bioinformatic pipeline.

**Results:**

After initial analysis, 441 loci had a nominal *P* value < 0.05 when examining the effect of ischemia *vs*. normal heart tissue on a normal diet in the absence of treatment. 426 loci at *P* value threshold < 0.05 were identified when comparing the ischemic *vs*. normal tissue from high-fat diet animals. When examining the effect of EV treatment in ischemic tissue in subjects on a normal diet, there were 574 loci with nominal *P* value < 0.05 with two loci Fructosamine 3 kinase related protein [(FN3KRP) (*P* < 0.001)] and SNTG1 (*P* = 0.03) significant after Bonferroni correction. When examining the effect of EV treatment in ischemic tissue in HFD, there were 511 loci with nominal *P* values < 0.05. After Bonferroni correction, two loci had *P* values less than 0.05, betacellulin [(BTC) (*P* = 0.008)] and [proprotein convertase subtilisin/kexin type 7 (PCSK7) (*P* = 0.01)].

**Conclusions:**

Alterations in DNA methylation were identified in pig myocardium after ischemic insult, change in diet, and treatment with EVs. Hundreds of differentially methylated loci were detected, but the magnitude of the effects was low. These changes represent significant alterations in DNA methylation and merit further investigation.

## INTRODUCTION

Cardiovascular disease is a major source of morbidity and mortality across the globe^[1]^. Within the United States, it is the leading cause of death, with an individual dying from cardiovascular disease every 33 s^[2]^. Given the burden of myocardial ischemia (MI) on society, a thorough understanding of the factors that contribute to its pathogenesis is essential to prevention, diagnosis, and early intervention.

The incidence of cardiovascular diseases is highly associated with genetic factors and environmental variables, such as diet^[3]^. More recently, antidiabetic medications, such as SGLT2 inhibitors, have demonstrated benefit in the treatment of heart failure in non-diabetic patients, resulting in a level 1A recommendation for their use in heart failure irrespective of diabetic status^[4]^. Given the number of patients suffering from both metabolic syndrome (MetS) and cardiovascular disease, elucidating the interplay between these two conditions is of the utmost importance. With the emergence of the field of epigenetics, recent studies have shown that the cardiovascular impact of environmental factors such as diabetes, malnutrition, smoking, and hypertension can be mediated by epigenetic changes, particularly through DNA methylation, histone modification, and alterations in microRNA^[5]^. Although the effects of a high-fat diet on cardiovascular outcomes have been reported^[6]^, the mechanism by which diet may influence the epigenome is not well understood.

In addition to hypertension and other cardiovascular diseases, a large number of studies have reported the role of epigenetic regulation in MI^[6,7]^. One animal study investigating genome-wide DNA methylation in a mouse model identified significantly different methylation at various 5′-C-phosphate-G-3′ (CpG) sites at 10 min, 1 h, 6 h, 24 h, and 72 h post acute MI compared to the sham group^[8]^. Several human studies have reported differential DNA methylation in blood cells or circulating EVs associated with cardiac ischemia^[9–11]^. Notably, one study reported a relationship between acute coronary syndrome (ACS) and blood DNA methylation at 47 CpG sites through a genome-wide analysis of DNA methylation of whole blood in ACS patients and matched controls^[9]^. Another study investigating DNA methylation with MI and matched controls found significant changes in DNA methylation at 34 CpG sites in gene foci related to the pathogenesis of MI, including lipid metabolism and inflammation^[10]^. However, this group did not investigate alterations to the epigenome of the myocardium itself.

Extracellular vesicles (EV) are small membrane-bound structures derived from cells that have been demonstrated to carry cellular products such as miRNA, cytokines, proteins, and growth factors^[12]^. Previous research from our lab has shown that treatment of ischemic myocardium with EVs derived from mesenchymal stem cells results in improved myocardial perfusion, increased vascular density, and upregulated expression of angiogenic markers^[12,13]^. Furthermore, EVs have been shown to alter the epigenetic landscape in several ways, from altering mRNA expression to altering histone methylation^[11,14]^. However, there remains a paucity of information regarding the effect of EV treatment on the methylome of ischemic myocardium.

These studies highlight the importance of investigating the molecular and genetic mechanisms underlying the physiologic and pathophysiologic response to cardiac ischemia. Here, we present a subgroup analysis from our prior swine EV studies on the epigenome-wide DNA methylation profiles of porcine myocardium after ischemic insult in the setting of treatment with EV therapy and in normal *vs*. high-fat diet pigs.

## METHODS

### Isolation and characterization of extracellular vesicles

In a technique previously reported by our lab, EVs were derived from human bone marrow-derived mesenchymal stem cells (Lonza, Allendale, NJ). Cells were cultured in MSCGM BulletKit media (Lonza) and incubated until reaching 80% confluence per manufacturers’ recommendations. After reaching the desired confluence, culture media was replaced with serum-free RPMI media for 24 h. Mesenchymal stem cell cultures then underwent ultracentrifugation (ThermoScientific Sorvall WX Ultra Series Centrifuge with a Sorvall Surespin rotor, ThermoScientific, Waltham, MA) at 100,000 g for 75 min before washing with sterile PBS and repeating ultracentrifugation at 100,000 g for 30 min in order to isolate EVs which were resuspended in 10% DMSO. For the administration of EVs to porcine myocardium, a solution of 50 ug of EVs suspended in 2mL of sterile 0.9% saline was created on the day of surgery^[12,13]^.

Confirmation studies of EV characteristics were undertaken and previously reported. In brief, qualitative proteomic analysis was performed and confirmed the presence of EV biomarker proteins “(CD44, CD81, CDC42, EGFR, CTNNB1, CAMK2D, CAMK2G, COL1A2 and FLNA)”^[15]^. Furthermore, morphological conformation via electron microscopy was undertaken, the results of which were previously published by our lab and can be viewed in citation 12, Scrimgeour *et al*. PLoS One. 2020 Sep 11^[12]^.

### Swine model of chronic cardiac ischemia

All animal procedures were conducted in accordance with protocols approved by the Institutional Animal Care and Use Committee of Rhode Island Hospital (#505821). We selected a well-characterized, high-fidelity, large animal model of chronic myocardial ischemia^[12,13,15–19]^. In a technique previously described by our lab, twelve 11-week-old Yorkshire swine were divided into two groups, either normal diet control (NDC) (*n* = 6) or high-fat diet (HFD) (*n* = 6). At 11 weeks of age, pigs were anesthetized with intramuscular telazol to facilitate endotracheal intubation by veterinary staff, at which point anesthesia was maintained with inhaled isoflurane. While maintaining sterile technique, a left thoracotomy was performed in order to expose the heart, then opened the pericardium and dissected out and isolated the left circumflex artery at the point of its takeoff from the left coronary artery. After isolation of the circumflex artery, an ameroid constrictor (Research Instruments SW, Escondido, CA) was placed around the left circumflex artery in order to induce chronic ischemia. The pericardium and thoracotomy were then closed in layers^[13]^.

### EV treatment

Two weeks after the placement of the ameroid constrictor, the swine underwent redo thoracotomy and injection of EVs (3 NDC and 3 HFD) or vehicle control (3 NDC and 3 HFD). For this operation, the same anesthesia protocols listed above were used. After anesthesia, a left thoracotomy was performed via an incision 2–3 cm below the site of the prior thoracotomy to avoid any interference with tissues that had adhered to the thoracic wall at the site of the prior incision. Once in the thoracic cavity, the pericardium was opened and the area of the left ventricle supplied by the left circumflex artery was visualized. Ten injections at various sites using a total injection volume of 50 ug of EVs suspended in 2 mL of sterile saline (3 NDC and 3 HFD) or vehicle control (3 NDC and 3 HFD) were performed in the area supplied by the LCx artery. At 19 weeks of age, all pigs were humanely euthanized and cardiac tissue was harvested^[13]^.

### Myocardial perfusion

In a protocol previously reported by our group at the time of terminal harvest, 5 mL of Lutetium and Europium labeled microsphere were injected into the left atrium. Simultaneously, 10 mL of blood was collected from the femoral artery. This procedure was then repeated under paced conditions at 150 bpm using Samarium-labeled microspheres. Post-harvest left ventricular tissues was weighed dried and sent to Biophysics Assay Laboratory along with the corresponding blood samples. Quantification of microsphere content in relation to blood flow could then be calculated for all myocardial segments^[13]^.

### DNA isolation

DNA was isolated from a 30 mg sample of homogenized ventricular tissue using a standard Qiagen DNeasy procedure per manufacturer’s instructions, and the quality and quantity of DNA was determined spectrophotometrically (Implen, CA, USA)^[20]^.

### Methylome analysis

DNA methylation profiling was performed via reduced-representation bisulfite sequencing (RRBS) (Epigentek, Farmingdale, NY, USA).

Bioinformatic analysis was performed at the Computational Biology Core, Center for Computation and Visualization of Brown University. Initial quality control of raw reads was performed using FASTQC^[21]^. Reads were then trimmed using Trim Galore and Trimmomatic^[22,23]^. Then, using Bismark, the reference genome was prepared and trimmed reads aligned^[24]^. Bismark methylation extraction could then be used to extract methylation information, at which point the edgeR package could be used to find differentially methylated loci (DML)^[25]^.

Loci were filtered to include chr1-18, X, and Y chromosomes, exclude loci that were never methylated or always methylated (as these provide no information about differential methylation) and include only CpGs with at least 1 count (methylated or unmethylated) across all samples. The resulting sample size after filtering was 68,998 loci.

For each lotus left after filtering, we calculated the distance to the nearest Ensembl gene using the distanceToNearest function in the GenomicRanges package. Methylated and unmethylated counts for loci that were within 2 kilobases of a gene were aggregated (summed), resulting in methylated and unmethylated counts across 7,770 gene promoters. The summed counts per gene promoter were used for downstream statistical analysis.

The glmFit function from edgeR was used to fit a negative binomial generalized log-linear model to test for differential methylation in gene promoters. The experimental design matrix was constructed using modelMatrixMeth from edgeR with a factorial experimental design (~0 + group), where group was a factor variable with levels comprised of each combination of treatment, diet, and tissue. We dropped the intercept from our model to parameterize it as a means model. This approach allowed us to build contrast vectors to find differentially methylated loci for specific comparisons of interest, which we performed using the glmLRT function from edgeR. Promoters were considered differentially methylated if the nominal *P*-value was < 0.05. Results were filtered based on this nominal *P*-value threshold.

Pathway analysis was performed using GeneGo Metacore (Clarivate, PA, USA). Metacore is a large curated database of known factor-to-factor interactions and allows designing networks based on these known “interactions”. The “direct interactions” algorithm thus builds a graphical network of factors from a list (without adding intermediaries) connected by interactive lines between them, each depicting a published interaction between the two factors.

Pathway mapping in Metacore relies on pre-designed graphical maps of specific pathways which form the pathway database. The algorithm determines what genes (factors) in the source list also occur in the graphically pre-designed existing pathway maps and selects the top pathways enriched.

## RESULTS

**Figure 1.**
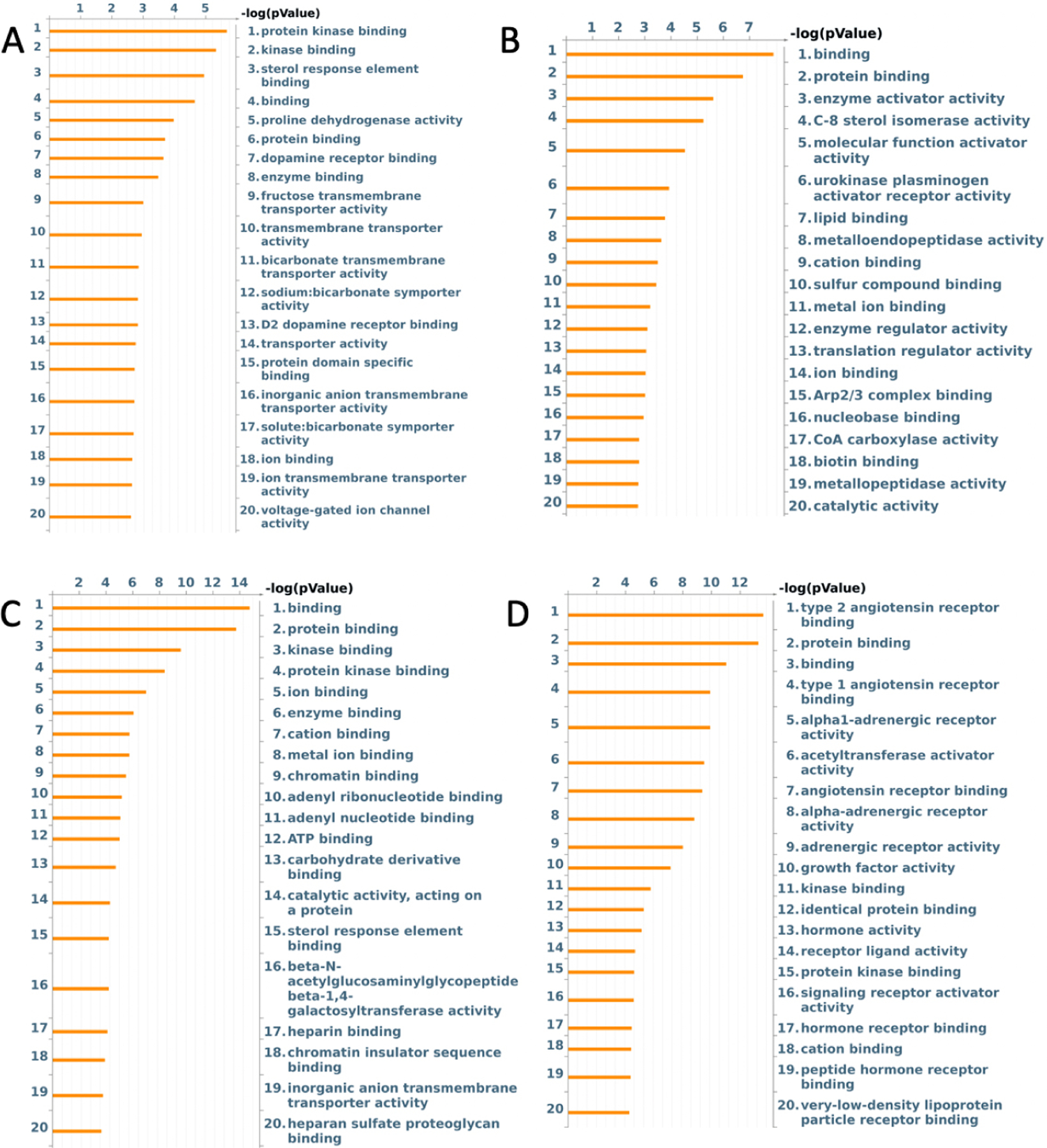
Enrichment analysis of molecular functions in (A) ischemia effect, (B) diet effect, (C) EV effect in normal diet, and (D) EV effect in HFD.

**Figure 2.**
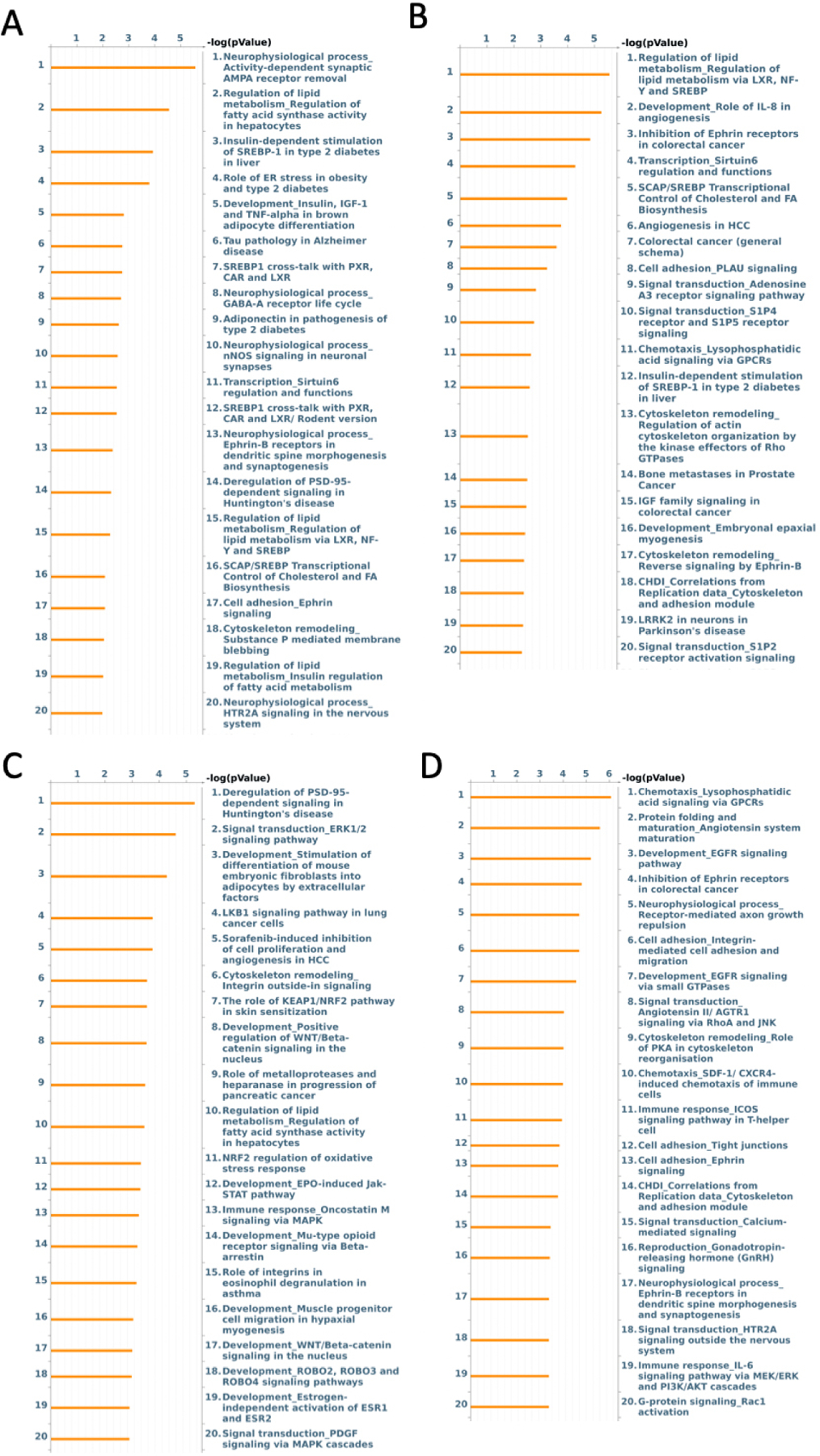
Enrichment analysis of molecular pathways in (A) ischemia effect, (B) diet effect, (C) EV effect in normal diet, and (D) EV effect in HFD.

After initial analysis, 441 loci had a nominal *P* value < 0.05 when examining the effect of ischemia *vs*. normal heart tissue on a normal diet in the absence of treatment (i.e., saline control perfused segment *vs*. saline control ischemic segment). When enrichment of these loci was performed using Metacore, the alterations in both molecular pathways and molecular functions were elucidated [Figure 1A and Figure 2A]. Specifically, alterations involved the molecular functions with terms “protein kinase binding”, “kinase binding”, “sterol response element binding”, and “proline dehydrogenase activity”. GoProcess annotation revealed that most of these effects were concentrated on processes related to “regulation of lipid metabolism”, “insulin-dependent stimulation of SREBP-1 in type 2 diabetes in liver”, and “role of ER stress in obesity and type 2 diabetes”. Top pathway names and related *P* values are shown in Figure 2A. After Bonferroni correction, no *P* values were < 0.05.

426 loci at *P* value threshold < 0.05 were identified when comparing the ischemic *vs*. normal tissue from high-fat diet animals. After undergoing enrichment analysis, the most significant change among molecular functions was associated with terms “binding”, “protein binding”, “enzyme activator activity” and “C-8 sterol isomerase activity” [Figure 1B]. After GoProcess annotation, the pathways identified with alteration in methylation were regulation of lipid metabolism, development role of IL-8 in angiogenesis, inhibition of ephrin receptors, and transcription sirtuin6 regulation and functions [Figure 2B]. Similar to the effect of ischemia in normal diet, after Bonferroni correction, no *P* values were < 0.05.

**Figure 3.**
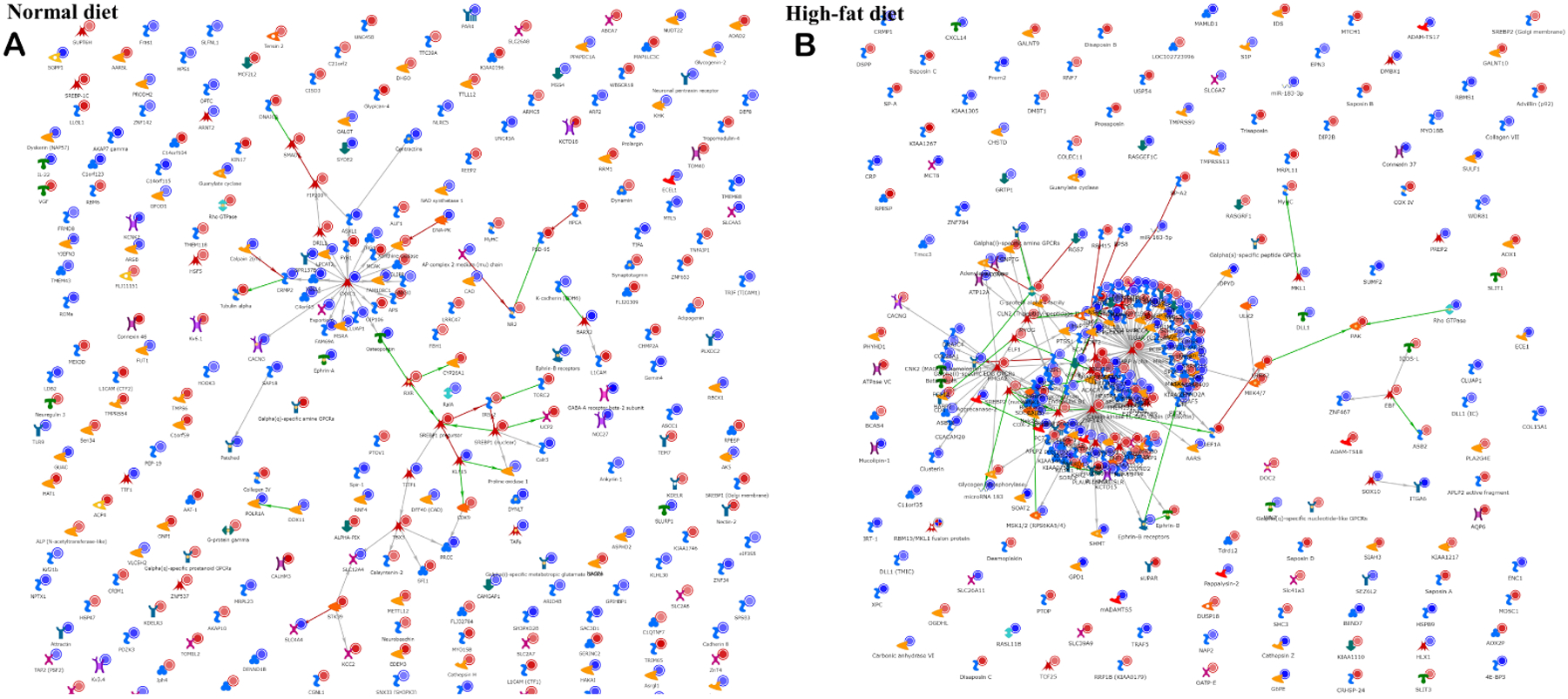
Network analysis. A “direct interactions” network tool in Metacore was used to plot known connections between the factors (genes) in a list of DMLs in a comparison of ischemic *vs*. normal tissue on (A) normal diet, or (B) high-fat diet background.

While the numbers of DMLs in the high-fat background and normal background were approximately the same, network analysis in Metacore [Figure 3] demonstrates a substantially more interacting network in the high-fat than in the normal diet.

When examining the effect of EV treatment on ischemic tissue under normal diet conditions, there were 574 loci with nominal *P* values < 0.05, with two loci FN3KRP (*P* < 0.001) and SNTG1 (*P* = 0.03) significant after Bonferroni correction. Enrichment analysis showed the molecular functions of “binding”, “protein binding”, “kinase binding”, and “ion binding” involved [Figure 1C]. Enrichment of molecular pathways revealed deregulation of “PSD-95-dependent signaling in Huntington’s disease”, “signal transduction ERK1/2 signaling pathway”, and “LKB1 signaling pathway in lung cancer cells” [Figure 2C].

When examining the effect of EV treatment in ischemic tissue in HFD, there were 511 loci with nominal *P* values < 0.05. After Bonferroni correction, two loci had *P* values < 0.05, BTC (*P* = 0.008) and PCSK7 (*P* = 0.01). After enrichment, most effects on molecular function were related to type “2 angiotensin receptor binding”, “protein binding”, “type 1 angiotensin receptor binding”, and “alpha1-adrenergic receptor activity” [Figure 1D]. After pathway enrichment, changes appeared to be most concentrated on pathways involving “chemotaxis”, “lysophosphatidic acid signaling via GPCRs”, “protein folding and maturation in angiotensin system maturation”, “development EGFR signaling pathway”, and “inhibitor of ephrin receptors” [Figure 2D].

**Figure 4.**
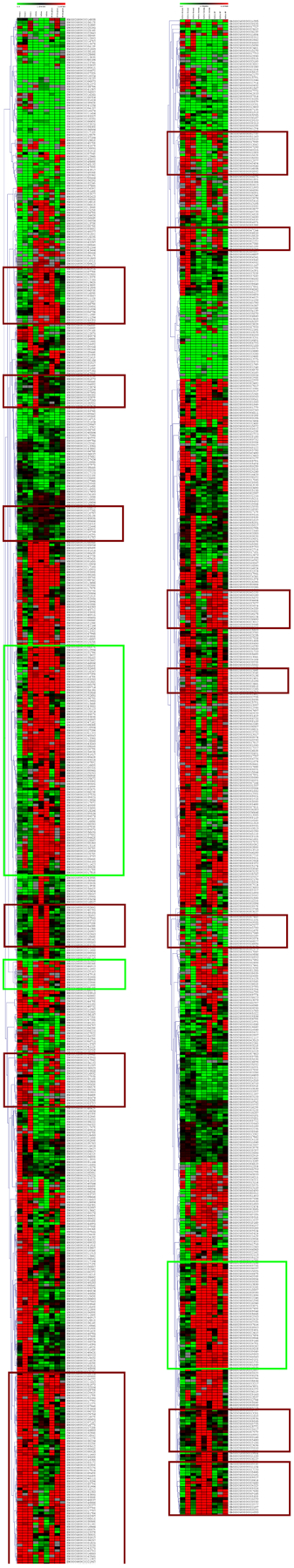
Hierarchical clustering of the loci differentially methylated in ischemia *vs*. control in the normal (left) and high-fat diet (right) reveals clusters where methylation was either increased (red) or decreased (green) in ischemia (3 samples in the middle) compared to control (3 samples on the left). These changes were nullified by EV treatment in some (brown rectangles) but not all (green rectangles) loci.

We employed cluster analysis to identify loci that were altered by ischemia compared to the control and then reverted by EV treatment. In both normal and high-fat diets, there were several such clusters (as highlighted by brown rectangle in Figure 4). This can potentially indicate the direct deleterious epigenetic effect of ischemia on cardiomyocyte methylome that is partly recoverable by EV therapy.

**Figure 5.**
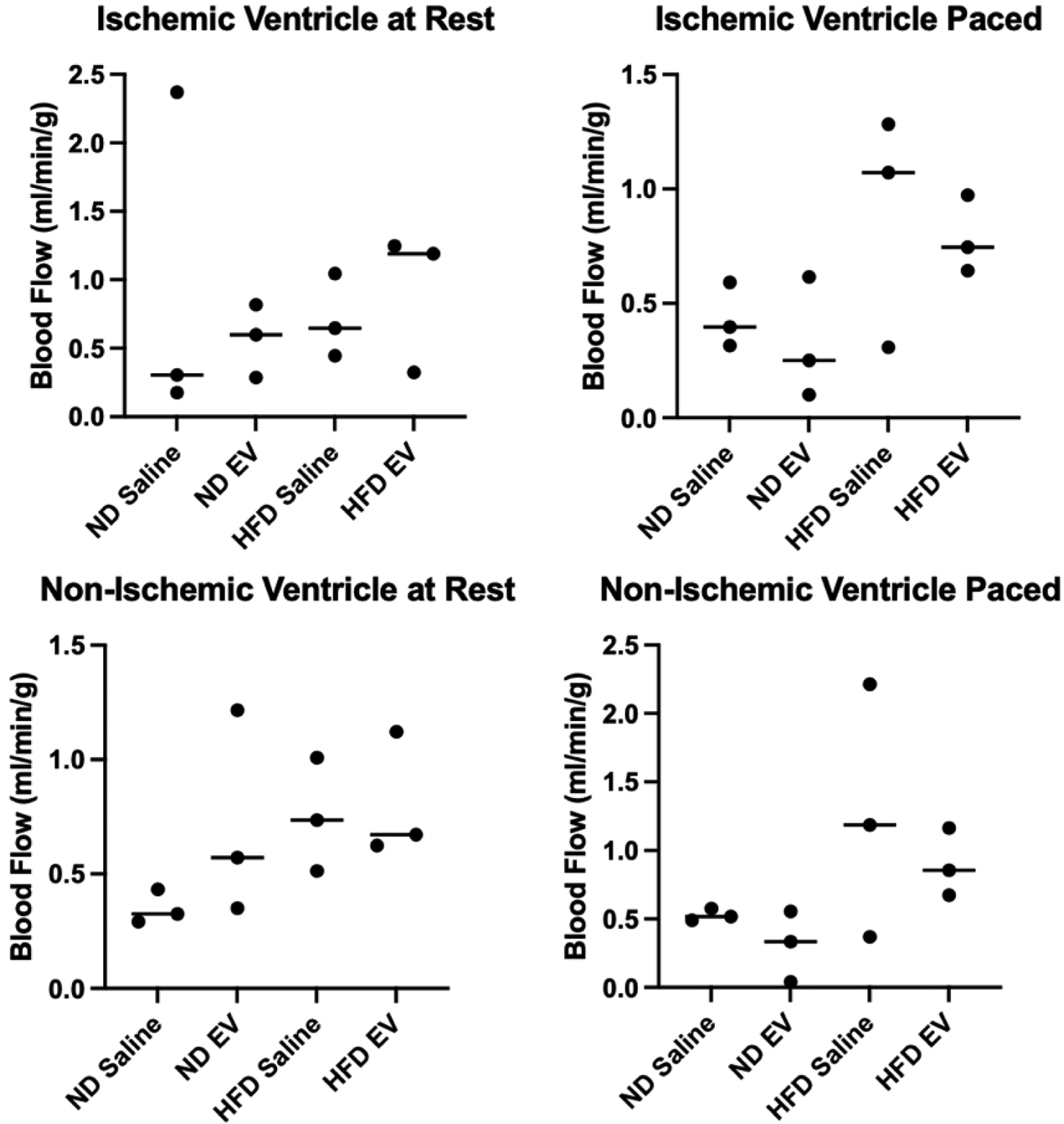
Measures of myocardial perfusion between 4 groups. EV therapy was not associated with any significant alteration to blood flow to ischemic myocardial territories under resting or paced conditions. Similarly, EV therapy was not associated with any significant alteration to blood flow to non-ischemic myocardial territories under resting or paced conditions.

While there were numerical differences between groups, there were no statistically significant differences in myocardial perfusion in either the ischemic myocardial segments or the non-ischemic segments either at rest and when paced [Figure 5].

## DISCUSSION

There is evidence of epigenetic change in response to cardiac ischemia. Cardiac events have been shown to significantly alter whole-genome methylation patterns in patients’ blood samples, and our own previous research has demonstrated the effect of cardiopulmonary bypass (CPB) on the methylome of skeletal muscle^[20,26]^. Alterations in the methylome have been associated with increased cardiac risk. For example, in the Framingham Offspring Study, alterations in DNA methylation were used to identify biomarkers associated with the risk of cardiovascular events^[27]^. Cardiac myocytes themselves have been shown to have altered epigenetic regulation with well-established changes in transcriptomic and proteomic profiles after ischemic events^[28,29]^. However, little is known about the alteration of the myocardial tissue methylome in response to ischemic stress. Given the extent to which the methylome has been shown to be altered in other physiologic and pathophysiologic states, investigating the role of tissue-specific methylation in ischemic cardiac tissue is of interest.

Our enrichment analysis of methylated loci suggests that in a normal diet, ischemia alone can induce a change in the methylome. By performing a pairwise comparison, we were further able to investigate the independent effect of a high-fat diet on epigenetic response to cardiac ischemia. It has been well established that metabolic syndrome alters the pathologic response to ischemic stress, and our results suggest that alteration in the cardiac methylome may be a part of this maladaptive response. Treatment with EVs appeared to have an independent effect on the methylome of normal diet swine and our swine model of metabolic syndrome.

Previous work using the same experimental animals that were used in this study demonstrated profound changes in ischemic myocardium in response to EV therapy. Immunofluorescent examination demonstrated increased expression of arteriolar marker, SMA, and endothelial marker, CD31, in ischemic myocardium treated with EVs compared to saline controls^[15]^. This increase in both arteriolar and capillary density prompted immunoblotting analysis of angiogenic signaling and revealed increased expression of VEGR1, MAP kinase, and phosphorylated-MAP kinase, while significant downregulation in VEGFR2 was observed^[12]^. Previously, we also undertook an investigation of transcriptional mRNA expression in chronically ischemic swine myocardium after EV treatment in HFD and normal diet swine. Interestingly, EV treatment in ND resulted in gene enrichment in many metabolic pathways, including aerobic respiration, electron transport chain, and oxidative phosphorylation, while differential expression in HFD EV-treated swine centered around immune pathways^[13]^.

In addition to the aforementioned observations previously published by our group, we have demonstrated multiple physiologic alterations in response to EV therapy. EV treatment improved cardiac output, stroke volume, and angiogenesis in our model of ischemic cardiac disease. Despite these profound effects, the full extent of the mechanism by which they work remains unclear^[16]^. These results suggest that further investigation of epigenetic alterations induced by EV injection is warranted to elucidate the mechanism behind their effect.

### Ischemia effect

When investigating the effect of ischemia, our results demonstrated changes in molecular functions and pathways involved in various metabolic pathways. Given the known metabolic shifts (i.e., glycolytic shift) in cardiac ischemic, alteration of DNA methylation may represent one mechanism of metabolic response to ischemia; however, more research is needed to determine this conclusively. Previous studies have shown consistent alteration in the methylome of blood samples of patients with ACS^[26,30]^. It has been previously established that MI can cause acute changes in the methylation pattern of DNA as early as 6 h post MI in mouse models^[8]^. Much like our enrichment analysis, prior studies have shown alteration in the biological processes of immune system processes, and regulation of responses to stimuli and cell activation after enrichment analysis^[26]^. We observed alteration in the molecular function of sterol response element binding and pathway analysis showed changes in regulation of lipid metabolism in our analysis. This is consistent with this existing literature, which has shown enrichment in phospholipid, protein, and lipid binding activities in patients with ACS^[26]^.

### Diet Effect

There have been well-established links between cardiovascular risk and diet^[31]^. This increased risk is multifactorial. There has been a well-established effect of diet on DNA methylation, most notably the work surrounding the individuals who survived the Dutch Famine and their offspring^[32]^. More recently, small animal models have shown alteration in the atherosclerotic response of hypercholesterolemic mice with altered histone methylation patterns in vascular tissue. Specifically, one example of this is alterations in the methylation of genes coding for eNOS, which have been implicated in atherosclerotic heart disease in humans^[33]^. Interestingly, as with the Dutch Hunger, these epigenetic changes appear to be stable and transfer to subsequent generations of mice^[33]^. While no individual gene reached significance, enrichment analysis of our nominal data revealed an altered methylation in pathways involved in lipid metabolism, and angiogenesis. Given that alteration of the methylome of these pathways has been implicated in multiple tissue types across a variety of animal modules of cardiovascular disease, their epigenetic regulation may represent a key area of investigation into the physiologic response to cardiovascular insult.

### EV effect in ND

Our analysis of the EV effect also demonstrated a significant alteration in methylation of the FN3KRP gene, which codes for a protein involved in the reversal of glycation, a process known to play a part in arterial stiffening^[34]^. It is possible that this housekeeping gene is methylated as a maladaptive response to ischemic stress and merits further investigation. Previous studies have investigated alteration in the methylation pattern of DNA in EVs of patients with ACS^[11]^. Furthermore, one group investigated the effect of ACS EVs on PMBCs and demonstrated that EVs did alter the DNA methylation profile of PMBCs. Enrichment analysis of pathways with altered methylation patterns included Wnt signaling pathway, cadherin signaling pathway, angiogenesis, and PI3 Kinase pathways^[11]^. Much like PMBCs altered by treatment with ACS EVs, we observed enrichment analysis of our EV-treated normal diet swine myocytes demonstrated alteration of the Wnt pathway [Figure 2C]. However, our analysis did not reveal any alteration to the cadherin signaling pathway, angiogenesis, or the PI3 kinase pathway, suggesting that myocytes and blood may not have the same epigenetic response to ischemic stress. Given this, we believe methylome alteration may be in part responsible for tissue-specific response to ischemic insult, though this will require further study.

### EV effect in HFD

Previously, our lab demonstrated an improvement in diastolic function and increased expression of eNOS, VEGFR2, and MAPK ERK1/ERK2 in an EV-treated MetS swine model of chronic ischemia^[13,15]^. Studies in mice have also demonstrated EVs can play a critical role in the pathogenesis of insulin resistance via alteration in AhR signaling^[35]^. Despite this, the exact mechanisms driving these changes remain unclear. In this study, within the EV HFD group, we were able to show alteration in methylation of the PCSK7 gene, which has been implicated in hyperlipidemia^[36]^. As hyperlipidemia is a known risk factor of cardiovascular disease and this gene was only shown to have a significant difference in methylation with EVs in HFD swine, epigenetic alteration via methylome manipulation may be a mechanism by which improvement in cardiac outcomes is seen in EV treated swine. Similarly, we observed significant methylation of the BTC gene, a member of the EGF family, and our enrichment analysis of genes found to be nominally alerted prior to Bonferroni correction showed alteration of multiple EGFR-related pathways. Given the known role of the EGF MAPK pathway in cardiac hypertrophy, this altered methylation of the BTC gene may yet again represent a mechanism by which the observed benefits of EV treatment may be explained. These alterations in the methylome invite an intriguing possible mechanism by which the observed effect of EVs is accomplished via epigenetic regulation; however, this will require further investigation.

### EV effect on myocardial perfusion

In the current analysis, which represents a subgroup that underwent methylome analysis of our whole swine cohort, we did not observe significant differences in myocardial blood flow in the HFD swine treated with EVs either at rest or during pacing. However, this finding is likely due to the small sample size used in this study [Figure 4]. Our previous research using an entire cohort that underwent the same protocol revealed a significant increase in perfusion at the rest of EV-treated swine in both normal diet and HFD groups compared with saline injection controls^[13]^.

### Limitations

Our study was not without its limitations. Given the small sample size of each arm (*n* = 3), we may have been underpowered to demonstrate significance. Given this lack of power, we may have also been vulnerable to type II error after the Bonferroni correction. This lack of power may have contributed to the similarity in the scope of detectable changes by our experimental conditions, and prompts further repeatability studies that could test this premise. The animal model itself also represents the obvious limitation of being a surrogate used to examine a chronic human condition and can only approximate the pathophysiology and physiologic response to disease seen in patients. However, this large animal model does allow for a closer examination of these processes and is likely more relevant than small mammal studies.

### Conclusion

Examination of the effect of diet and EV treatment on the methylome of chronically ischemic cardiac tissue has raised intriguing possibilities regarding the epigenetic response to ischemic stress. This investigation further demonstrates that not all tissue has the same epigenetic response to a stressing event, meaning that blood may not be an accurate surrogate for events occurring in the myocardial methylome. Although our study did not reveal a global change or singular response by the entire methylome, this is likely due to a lack of statistical power. Nevertheless, it is noteworthy that responses in specific methylation patterns may represent a subtler, yet still impactful, tissue response to ischemic stress.

## Data Availability

All methylome data has been submitted to GRA/GEO, and all additional data will be made available upon reasonable request to the authors.
